# Combination therapy for pediatric patients with Kasabach–Merritt phenomenon: A single-center retrospective study

**DOI:** 10.1097/MD.0000000000030296

**Published:** 2022-08-26

**Authors:** Li Zhang, Lang Liu, Huanmin Luo, Wenbiao Xu, Huishan Chen, Muxia Yan, Yiqian Wang

**Affiliations:** a Department of Hematology, Guangzhou Women and Children’s Medical Center, Guangzhou Medical University, Guangzhou, China; b Department of Interventional Radiology and Vascular Anomalies, Guangzhou Women and Children’s Medical Center, Guangzhou Medical University, Guangzhou, China; c The Third Clinical School of Guangzhou Medical University, Guangzhou, China; d Department of Radiology, Guangzhou Women and Children’s Medical Center, Guangzhou Medical University, Guangzhou, China; e Department of Allergy, Immunology and Rheumatology, Guangzhou Women and Children’s Medical Center, Guangzhou Medical University, Guangzhou, China; f Department of Biochemistry and Molecular Biology, GMU-GIBH Joint School of Life Sciences, Guangzhou Medical University, Guangzhou, China

**Keywords:** embolization, hemangioma, Kasabach–Merritt phenomenon, sirolimus, steroid therapy

## Abstract

This study aimed to in the management of Kasabach–Merritt phenomenon (KMP), a severe thrombocytopenic coagulopathy that occurs in the presence of an enlarging vascular tumor. Here, we retrospectively evaluated 12 patients with KMP in Guangzhou Women and Children’s Medical Center, Guangzhou Medical University, from 2017 to 2021. 12 patients, including 7 females and 5 males, were identified. Tumors were located in the leg (n = 4), neck (n = 1), face (n = 3), chest wall (n = 1), back (n = 2), and retroperitoneum (n = 1). A plaque-like lesion with ecchymosis was the most common cutaneous manifestation. All the patients underwent embolization therapy. Nine patients received steroid treatment and 7 patients were administered with sirolimus. The mean duration of treatment was 1.6 months. All the patients reported in this study were alive when discharged. Embolization combined with steroid and sirolimus appears effective in patients with KMP, as well as in those who experienced disease recurrence. However, a long-term follow-up of the children cured of KMP will be necessary to monitor its recurrence and improve the outcome.

## 1. Introduction

Kasabach–Merritt phenomenon (KMP) is a coagulopathy usually caused by kaposiform hemangioendothelioma (KHE) and characterized by thrombocytopenia, microangiopathic hemolytic anemia, and a rapidly enlarging vascular tumor in either infants or young children.^[1–3]^ KMP usually presents as an enlarging, purpuric lesion, involving the trunk, extremities, face, and retroperitoneum.^[4]^ Despite the discovery of new therapies, there is no consensus treatment for it, and KMP is still associated with high morbidity and mortality.

As a first-line treatment, corticosteroids are considered an important reagent for treating KMP patients. In the past few decades, new treatments have emerged including vincristine, chemotherapy, and embolization. For example, it has been reported that the vascular endothelial growth factor R3-PI3K-AKT-mTOR signaling pathway is aberrantly activated in KHE or KMP, and sirolimus, an inhibitor of the mTOR pathway, has been proven to be effective and safe in clinical trials in childhood KMP.^[5]^ In addition, arterial embolization has been used in the past to rapidly reduce the tumor mass, which, on the other hand, carries the risk of necrosis around the lesion.^[6]^ Under extreme conditions, such as active bleeding, plate transfusion or cryoprecipitate would be adopted to avoid symptomatic anemia.^[7]^ However, standardized treatment strategies for KMP have not yet been established.^[8]^ Therefore, developing a better understanding of the clinical features and treatment for KMP is important to develop more effective treatment protocols.

In our study, we analyzed the clinical features and responses to treatment of 12 patients with KMP admitted to Guangzhou Women and Children’s Medical Center in China from November 2017 to January 2021. The aim of our study is to describe the clinical course of the patients before and after the combination treatment, which will hopefully guide future treatments for pediatric KMP.

## 2. Materials and Methods

A retrospective review was performed on 12 pediatric patients diagnosed with KMP associated with vascular tumors and treated in Guangzhou Women and Children’s Hospital in China between 2017 and 2021. The following information was collected retrospectively: patient age at diagnosis, gender, cutaneous manifestation, clinical presentation, anatomic location, platelet count, treatment, clinical course, and outcome. Initial diagnostic management included ultrasound, and magnetic resonance imaging for all cases. Deep lesions infiltrated retroperitoneal sites. Diagnosis of KMP was based on severe thrombocytopenia (< 50 × 10^9^/L). Therapeutic methods include steroid therapy (dexamethasone, XMS, 0.75 mg/kg; prednisone, 5 mg/kg), arterial embolization, propranolol (1 mg/kg), and sirolimus (0.05 mg/kg), depending on individual responses to treatment. Treatment response was based on plate count and classified as either good response (increase of > 50% of platelet count), poor response, or no response.

The research has been approved by Guangzhou Women and Children’s Medical Center’s appropriate ethics committee. Written informed consent was obtained from all parents or guardians according to institutional guidelines.

## 3. Results

### 3.1. Patient characteristics and clinical manifestations

**Figure 1.**
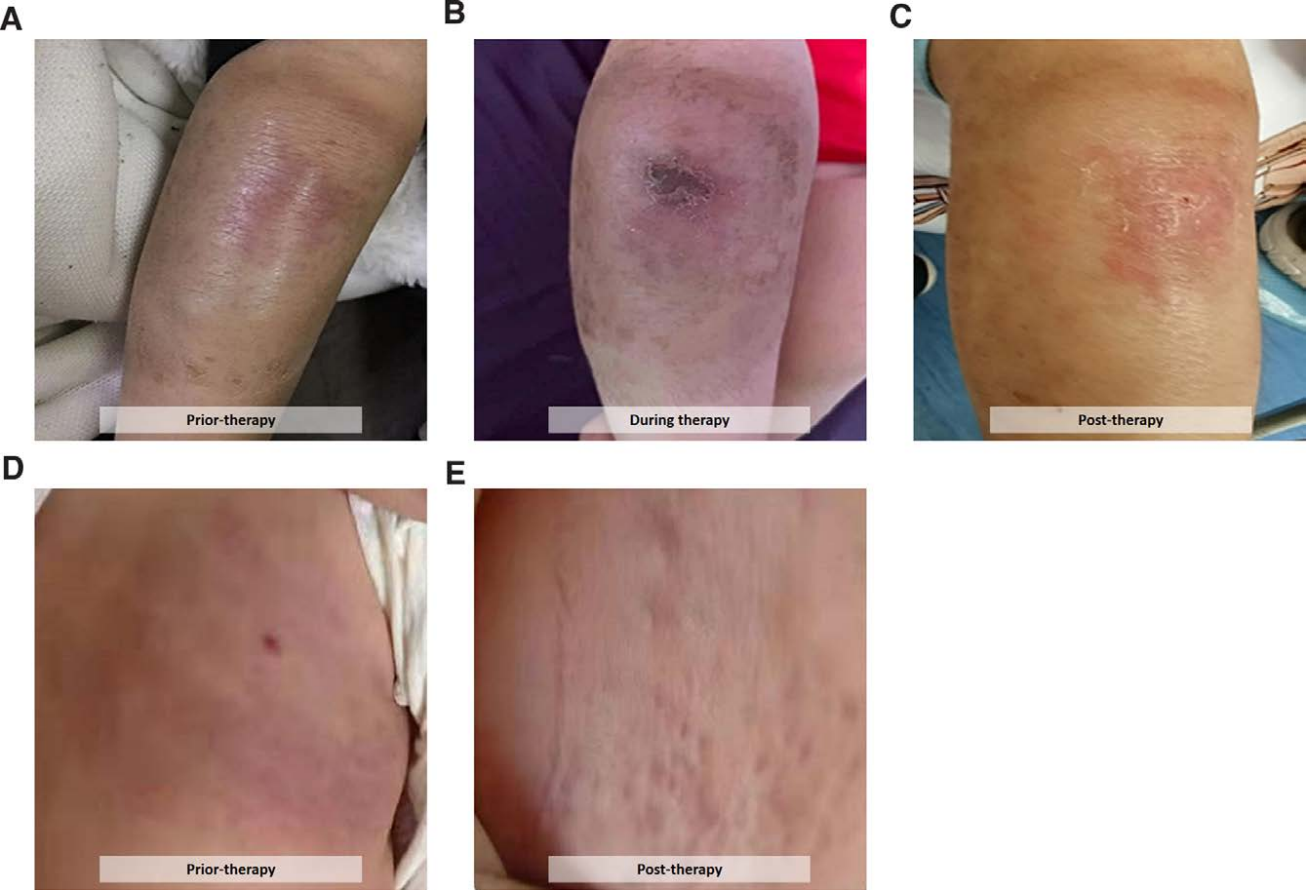
Representative clinical images of 2 pediatric patients with KMP before and after treatment. (A–C) Patient 11 had cutaneous hemangiomas on leg. (D, E) Patient 12 with cutaneous lesion on back responded well to the therapy with improvement in the appearance and size of the lesion. KMP = Kasabach–Merritt phenomenon.

We identified a total of 12 children who were diagnosed with KMP in our institute. Table 1 lists the clinical characteristics of all the patients. The mean age of diagnosis was 3 mo (range 1–6). Six children (58%) were females, and 4 (42%) were males (female to male ratio 3:2). Of note, 1 patient was born prematurely (38 weeks of gestation). The characteristics of the study population are summarized in Table 1. Of the 12, 11 patients had cutaneous hemangiomas with a plaque-like lesion with ecchymosis. Tumors were present in the leg (n = 4), neck (n = 1), face (n = 3), chest wall (n = 1), and back (n = 3). One patient had visceral hemangioma with a pancreatic hemangioma. The response rate for the patients reported in this study was 100% (Fig. 1).

**Table 1:** Patient characteristics.

n	Gender	Lesion location	Mean age at diagnosis	Overall survival
12	7 F (58%)	Leg (4)	3 mo (range, 1–6)	12 (100%)
	5 M (42%)	Neck (1)		
		Face (3)		
		Chest wall (1)		
		Back (2)		
		Visceral (1)		

F = female, M = male.

### 3.2. Treatment

Most patients reported in our current study had been treated heavily prior to their transfer to our institute. Remarkably, all the patients underwent embolization. Nine patients received steroid therapy, including XMS and prednisone, during the treatment process. Eleven patients received sirolimus. The details of the clinical characteristics of all the patients are shown in Table 2.

**Table 2:** Clinical manifestations.

Patient	Age (mo)/gender	Lesion location	Clinical characteristics	Treatments	Outcome
1	6/F	Left leg	Leg enlargement (5 × 6 cm), purplish lesion, erythema with clear border	Steroid, embolization, sirolimus	Free of disease
2	3/M	Neck	Neck enlargement, pain, red lesion	Sirolimus, embolization	Free of disease
3	2/M	Chest wall	Chest wall enlargement (10 × 8 cm), purplish lesion with ecchymosis, increased local skin temperature	Steroid, embolization, sirolimus	Free of disease
4	1/F	Face	Face enlargement (15 × 13 cm), purplish lesion with ecchymosis, maxillary and temporal osteolysis, increased local skin temperature, respiratory distress syndrome	Propranolol, steroid, embolization	Free of disease
5	2/F	Face	Face enlargement, purplish lesion	Propranolol, steroid, embolization, sirolimus	Free of disease
6	5/F	Left leg	Leg enlargement	Steroid, sirolimus, embolization	Free of disease
7	3/F	Visceral	Abdominal distension, bloody stools, cavernous transformation of the portal vein, hepatic bile duct dilatation, pancreatitis, a large number of ascites	Propranolol, steroid, embolization, sirolimus, sclerotherapy	Free of disease
8	1/M	Right leg	Leg enlargement, cutaneous and sclera icterus, respiratory distress syndrome	Steroid, propranolol, embolization	Free of disease
9	1/M	Face	Face enlargement, purplish lesion	Steroid, embolization, sirolimus	Free of disease
10	4/F	Back	Back enlargement, red lesion (3 × 3 cm)	Propanolol, steroid, embolization	Free of disease
11	6/M	Right leg	Leg enlargement, purplish lesion	Steroid, embolization, sirolimus	Free of disease
12	2/F	Back	Back enlargement, red lesion with obvious swelling	Steroid, embolization	Free of disease

F = female, M = male.

**Figure 2.**
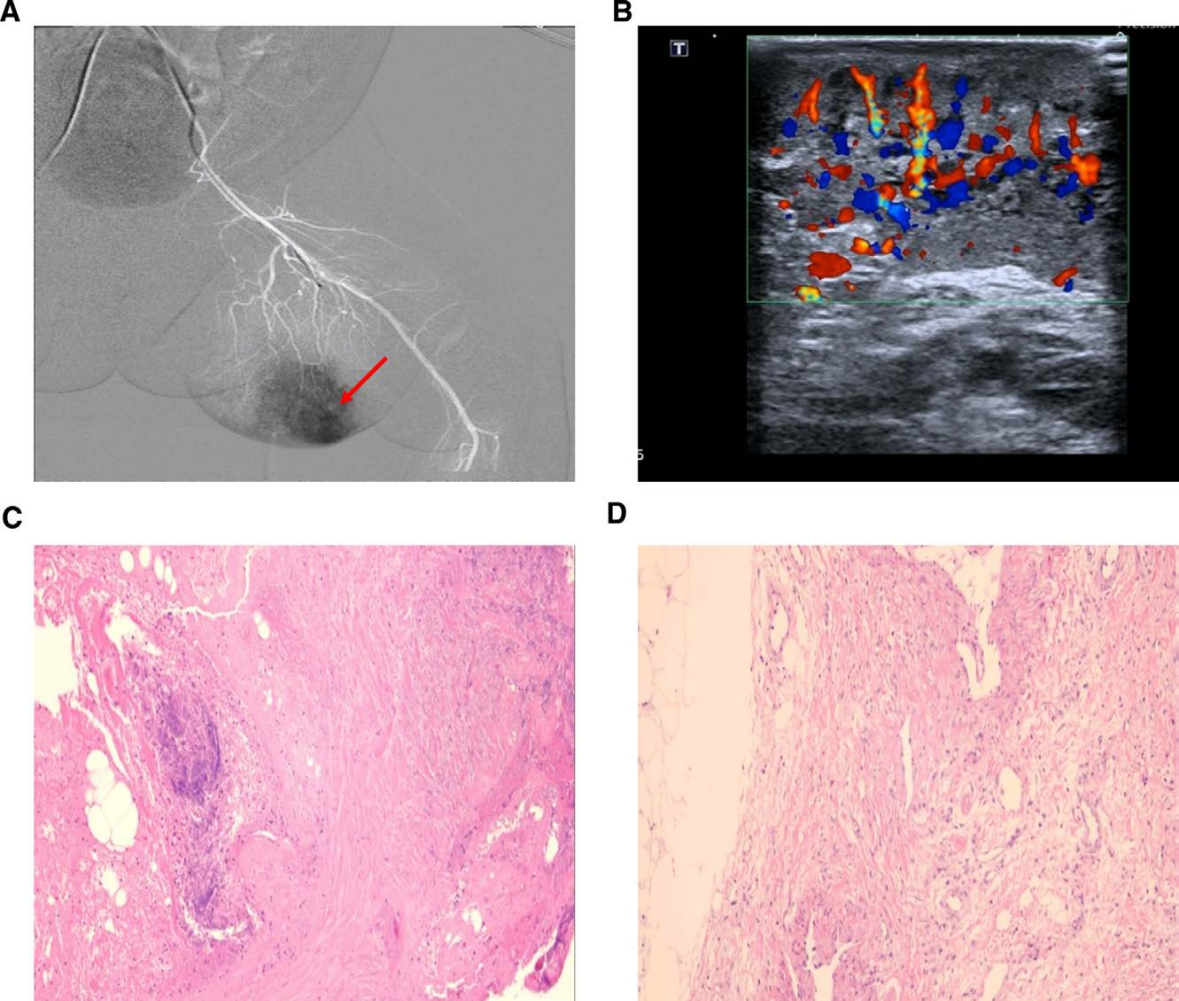
Representative clinical images of patient 1. (A) DSA detected a hypervascular lesion on the leg. (B) Ultrasound showed an infiltrative hemangioma of the lesion. (C, D) H&E staining revealed partial vascular hyperplasia, expanded and irregular-shaped lumens with thickened wall lined with a single layer of flat epithelial cells (400×). DSA = Digital subtraction angiogram, H&E = Hematoxylin and eosin.

**Figure 3.**
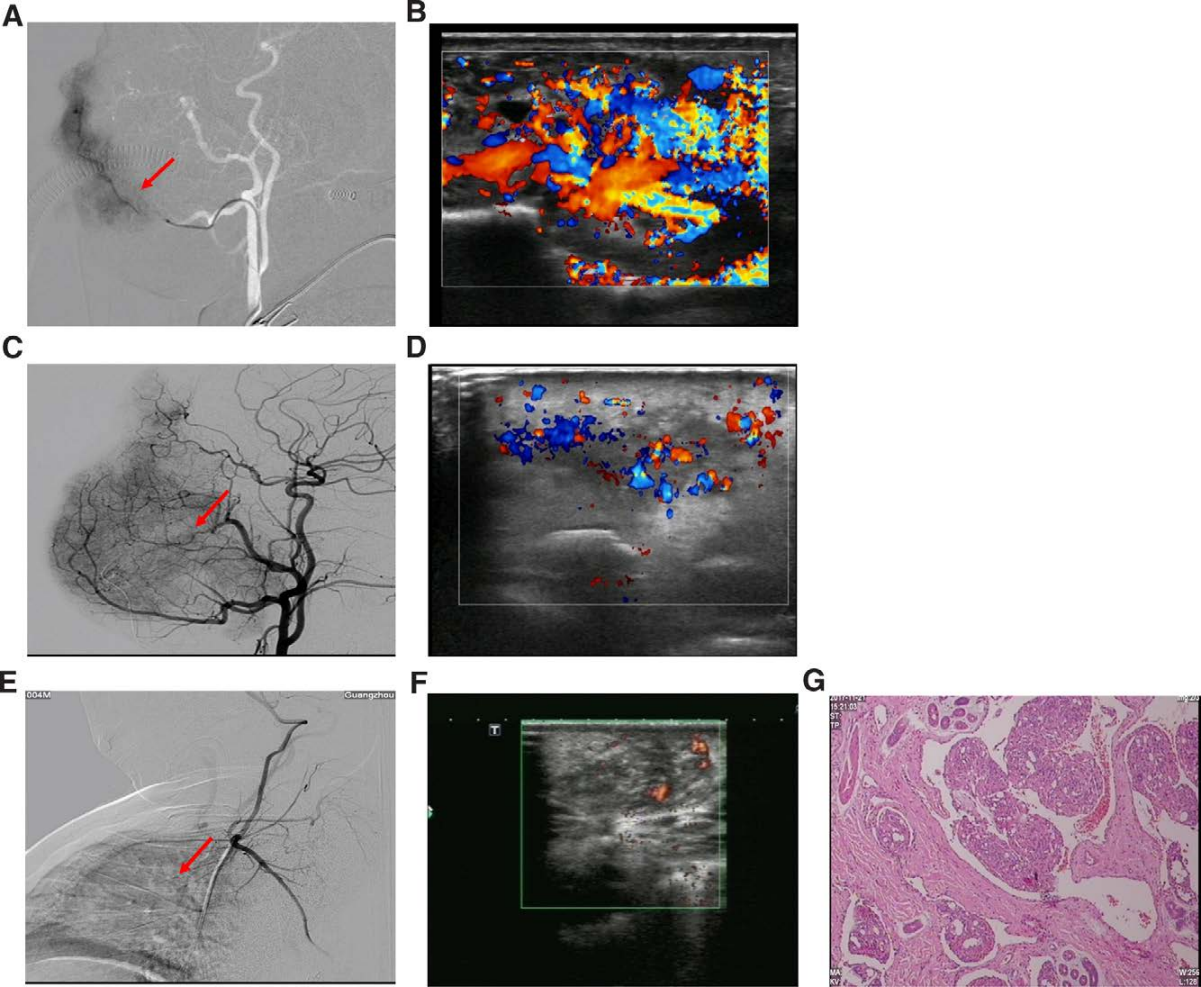
Representative clinical images at diagnosis. (A, C) DSA detected a hypervascular lesion on the face of patient 4 and 5. (B, D) Ultrasound showed an infiltrative hemangioma of the lesion of patients 4 and 5. (E, G) Representative DSA, ultrasound images of patient 10 at diagnosis, as well as H&E staining showing infiltrative lobules with spindle-shaped cancer cells (400×). DSA = Digital subtraction angiogram.

Patient 1 received steroid therapy, and the platelet count was increased. Subsequently, she had embolization in addition to sirolimus treatment (Fig. 2A–D). Patients 5, 7, and 8, having platelet counts of <20 × 10^9^/L, received platelet transfusions. Notably, patients 4, 7, and 8 who were diagnosed with a fibrinogen level of <200 mg/dL were administered with plasma infusion. Notably, patient 4 was identified with a deep-tissue face lesion despite having had continuous positive airway pressure therapy for respiratory failure during her previous hospitalization. The patient was transferred to our institute due to recurrence of KMP, where she was given XMS therapy and subsequently underwent arterial embolization. The coagulation function was restored with an enhanced platelet count after 4 weeks of treatment, and KMP did not recur (Fig. 3A, B). Patient 5 received propranolol, small doses of steroid, and platelet transfusion before being admitted to our hospital without obvious normalization of the platelet count or a resolution of the lesion. After being transferred to our institute, the patient was started on XMS and sirolimus following embolization. She finally showed better results, with considerable increase in the platelet count (Fig. 3C, D). Patient 8 was born prematurely and found to have jaundice on the skin and sclera. There was an improvement in the extent and appearance of the lesion after the patient had platelet transfusion, steroids, and propranolol therapy. However, the patient still had icterus. His platelet count restored to normal after he was moved to our institute and treated with arterial embolization. Patient 10 initially received propranolol, but her condition did not improve. After being transferred to our institute, we chose steroid pulse therapy and embolization to alleviate the symptoms of KMP (Fig. 3E–G).

**Figure 4.**
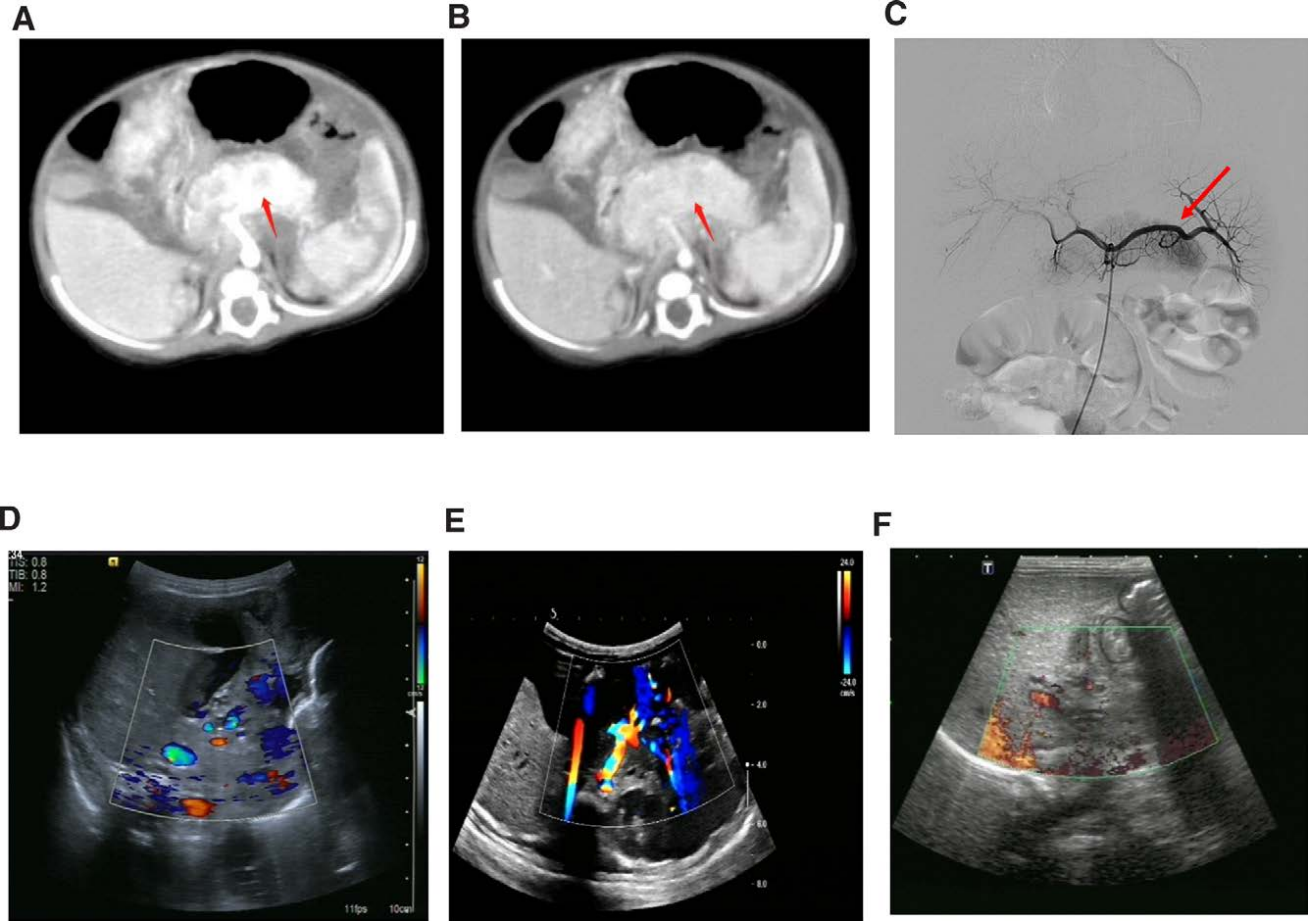
Representative clinical images of patient 7 before and after treatment. (A, B) Enhanced CT angiogram revealed a moderate enhancement in the retroperitoneum, demonstrated by the mass in the arterial phase and the venous phase at diagnosis. The arrow indicates the pancreas. (C) Based on the DSA result at diagnosis, there was no apparent tumor observed; however, there was a partial enhancement on the pancreatic tail. (D) Ultrasonographic findings of pancreatic hemangioma with increased peripheral echogenicity and hypoechoic central appearance. (E, F) No obvious arterial flow was detected after treatment in the pancreatic area. CT = computed tomography.

During her prior hospitalization, patient 7 suffered a gastrointestinal hemorrhage with abdominal distention and disseminated intravascular coagulation. Her bone marrow (BM) puncture results indicated that there was BM hyperplasia. The upper abdominal computed tomography showed the cavernous transformation of the portal vein, expanded biliary ducts, pancreatitis, edema and thickening of the intestinal wall, and ascites. The patient began fasting, received platelet transfusion, and adopted anti-infection treatment. After establishing the diagnosis of pancreatic hemangioma, the patient was treated with arterial embolization and sclerotherapy. Imaging of the pancreatic lesion showed enhancement with dense staining, which disappeared after the surgery. Following the surgery, the patient received sirolimus and steroid therapy and was subsequently cured of the ailment (Fig. 4A–F).

### 3.3. Outcome and follow-up

All patients are alive with a median follow-up of 2 years. Of note, patient 4 left the hospital with a normalized platelet count, while the lesion (3 × 5 × 1.0 cm^3^) on her right face was palpable with a clear boundary. Importantly, patient 7 showed no enhancement with deep staining anymore after embolization and sclerotherapy. The other patients had significantly smaller lesions, pain, or bleeding after treatment. No neurotoxicity or recurrence of the disease was observed during primary follow-up. However, a long-term follow-up study of the patients will be required to gain accurate information on the effectiveness of the treatments given to all of them.

## 4. Discussion

KMP has been thought to be caused by platelets trapped in the tumor by proliferating vascular endothelial cells, which are often characterized by thrombocytopenia and consumptive coagulation dysfunction, as well as tumors with ill-defined borders.^[1,7–10]^ The lesions in KMP patients usually appear in the form of purpuric masses on the surface, along with edema.^[11,12]^ However, lesions can also involve intrathoracic or retroperitoneal structures, which are often associated with higher mortality due to their delayed diagnosis.^[13]^ Therefore, improving our understanding of the diagnosis and treatment of KMP is of utmost importance in order to standardize the clinical treatment.

Propranolol is a nonselective β-adrenergic antagonist with different efficacies in the treatment of KMP.^[14,15]^ In our reports, patients 4, 5, 8, and 10 were administered with propranolol at a dose of 1 mg/kg, which was divided into 2 oral administrations. However, only patient 8 responded well, while patients 4, 5, and 10 ended with a larger hemangioma. They then underwent combination treatment, including steroid therapy and embolization, which caused a significant decrease in the tumor size and also improved their platelet count. Our results indicate that the therapeutic effect of propranolol was unsatisfactory in nearly three-fourth of the patients, implying that more research on propranolol being used as a treatment for KMP would be necessary before it is used as the first-line therapy.

Although the surgical approach is not considered as the first-line therapy for KMP patients, it is highly recommended when the effects of other drugs are not obvious, especially in cases when the lesion is extensive.^[16]^ Embolization has been widely applied for achieving a complete occlusion in the vascular tumor, including in patients with KMP.^[17]^ In our case, all of the 12 patients had received arterial embolization in combination with steroid therapy or sirolimus, and they were ultimately, free of disease. Our results indicate that, when necessary, glucocorticoids should be administered along with surgical procedures, implying that the adoption of systemic drug treatment along with embolization appears to be a safe treatment strategy in the case of life-threatening hemangioma in infants or young children with KMP.

Sirolimus has been previously reported to be effective in treating KMP patients, as it results in an increase in the platelet count and a decrease in the tumor size.^[18]^ Our patients’ responses to sirolimus as described above was rapid in terms of platelet count and coagulation function, especially when it was combined with steroid and embolization therapy. In a phase II clinical trial, Adams et al^[5]^ demonstrated the effectiveness of sirolimus in treating pediatric patients with complicated vascular anomalies without apparent BM toxicity. A more recent report by Harbers et al^[19]^ suggested that low-dose sirolimus successfully cured therapy-resistant patients with congenital vascular malformation, although adverse events such as menstrual cycle disturbances were observed in young adult patients. In addition, Sirolimus does have side effects, including immune suppression, hyperlipidemia, and oral ulcers. Thus, additional studies need to be performed to address those questions.

It is noteworthy that 2 patients in our study experienced disease relapse after recovery. These observations indicate that the “cure” for KMP lesions is not always completely permanent. Schaefer et al^[20]^ reported that 2 infants with KMP developed recurrence after they were discharged asymptomatically. Thus, our study emphasizes the need for a long-term follow-up to monitor pain or recurrence in patients with KMP. Follow-up of infantile patients with KMP for at least 1 year would be important to monitor pain and recurrence. More importantly, given the rarity of KMP, large collaborative prospective studies are essential to identify more effective treatment protocols for this life-threatening illness.

## 5. Conclusion

Taken together, our patients were promptly treated after diagnosis of KMP based on institutional experience. Additionally, it was observed that combined embolization, steroids, and sirolimus tend to be more effective in treating neonates with KMP. Moreover, it was also noted that long-term surveillance of patients after recovery is critical for ensuring higher survival rates. The ultimate goal of our work is to give new insights into treating KMP patients and modulate future clinical trials.

## Author contributions

LZ, MY, and YW conceived and designed the experiments. LZ, LL, WX, HC collected the clinical data and analyzed the data. LZ, MY, and YW wrote the manuscript. HL analyzed the data and participated in revision of the manuscript. All authors reviewed the manuscript.
